# Plastic, microplastic, and the inconsistency of human thought

**DOI:** 10.3389/fpubh.2023.1145240

**Published:** 2023-06-05

**Authors:** Antonio Ragusa, Caterina De Luca, Emma Zucchelli, Denise Rinaldo, Alessandro Svelato

**Affiliations:** ^1^Department of Obstetrics and Gynecology, Campus Bio-Medico University Hospital Foundation Rome, Rome, Italy; ^2^Department of Obstetrics and Gynecology, Fatebenefratelli Gemelli Hospital, Isola Tiberina, Rome, Italy; ^3^Instituto de Salud Global, Universitat de Barcelona, Barcelona, Spain; ^4^Department of Obstetrics and Gynecology, Azienda Socio Sanitaria Territoriale (ASST) Bergamo Est, Bolognini Hospital, Seriate, Italy

**Keywords:** human placenta, microplastics, raman microspectroscopy, human decisions, cognitive biases, plastic manufacturing industry

“*Mutability of temper and inconsistency with ourselves is the greatest weakness of human nature.”*

*seph Addison*

## Introduction

The influence that environment has on gestation is without a doubt underestimated. Despite being equipped with great intelligence, mankind possesses some significant cognitive limits that prevent the correct evaluation of events and their correlation to the distant future.

It is challenging to measure human intelligence as it is subject to numerous interdependent variables (1–7), some of which are as follows:

- Good nutrition;
- Regular schooling and school quality;
- Fortification of certain food products;
- Laws establishing safe levels of pollutants;
- Musical training in childhood;
- Socioeconomic status; and
- Incidence of infectious diseases.

Even more challenging, if it is possible, is to compare human intelligence with that of other species (8, 9). We can agree with Philip Sopher when he argues that “with the current infrastructure, analyzing animal intelligence is nearly impossible” (10). In this regard, we could just consider how underwhelming our olfactive intelligence is when compared, for instance, to the olfactive capacities of a dog, which is able to recognize and identify thousands of different smells. Adjectives like “smart” and “dumb” are irrelevant in cross-species comparisons.

Mankind systematically overlooks environmental data and the dramatic analytical data published by current scientific investigations (11, 12).

The way in which humans treat plastic may be considered a good paradigm of what we call “the inconsistent human thought.”

In the last century, we observed all over the world an exponential growth in the global production of plastic, which has crossed over 350 million tons per year. This is one of the largest contributors to environmental pollution (13). Due to the recent COVID-19 pandemic, we have seen a peak in discardable plastic production and consumption, primarily for personal protective equipment such as masks, scrubs, and gloves. Increased home deliveries caused an increase in non-recyclable plastic waste (14). Furthermore, unexpectedly and unscrupulously, big corporations, which recorded a reduction in fossil fuel sales, exponentially increased virgin plastic production (15).

Microplastics (MPs), defined as particles of < 5 mm, are derived mostly from plastic degradation in the environment (16). They are found in all the waters, oceans, lakes, rivers (17), soil (18), air (14), and food (19), especially in seafood (20, 21), sea salt (22, 23), and potable water (24, 25).

MP contamination can result from prolonged exposure, as well as after a single exposure (26, 27). There are three main pathways through which tiny MP particles may be absorbed: ingestion, inhalation, and dermal contact (28). To penetrate tissues, the particle diameter is usually ~5–10 microns (29), but particles with a larger or smaller diameter can also enter the human body (28). Depending on their dimensions, it is hypothesized that particles can spread through passive diffusion or active phagocytosis (30).

MPs were originally described in sea animals' gastrointestinal tract (31). Scientists were then able to demonstrate micro- and nano-plastic cellular absorption and accumulation in organic tissues (32–35) and human blood (36).

Our group published studies conducted on human samples, showing MPs in the placenta (29). MPs have also been found in newborns (37), thus suggesting contamination since the earliest stages of life. These findings are a worthy concern, and more studies are needed to investigate the direct consequences of MPs on human health. Are microplastics harmful? In animal models, toxicity has been reported. Studies demonstrated MP interference with energy production, lipid metabolism, increased oxidative stress, and neurotoxic responses (38). MPs seem to exert toxicological effects on cell cultures, causing apoptosis, inflammation, mitochondrial and lysosomal dysfunction, and genotoxicity (28). They may interact in a disruptive way with the immune system, altering the expression of genes involved in the immune response on a genetic level.

In mice models, MPs can determine changes on a phenotypic level in gene expression and epigenetics, as was demonstrated by brain abnormalities in mouse pups, whose mothers were fed with plastic microparticles (39). Cognitive capacity alterations were discovered, showing an alteration of RNA expression, and MP infiltration was observed through immunofluorescence in the brain of pups.

If microplastics interact with human placental cells altering energy pathways as described in animal models, there could be numerous concerning consequences (40).

Recently, our group published a study that demonstrates for the first time MPs in intracellular compartments of the human placenta (41). Moreover, we were able to localize microplastics and link their presence with important morphological and structural alterations of the cellular intracytoplasmic human organelles (41).

Using variable pressure scanning electron microscopy (SEM) and transmission electron microscopy (TEM), we detected MPs within lipid membranes. They can be easily confused with cell organelles such as lysosomes, peroxisomes, lipid droplets, and multivesicular bodies. These have never been seen before even though many scientists study placentas, but there is a great difference between “looking” and “seeing”. This amply confirms Goethe's [1749–1832] acknowledgment: “We only see what we know.”

In all the observed samples, the stress of the endoplasmic reticulum is evident, seen as being dilated (cribriform aspect of the syncytium trophoblast cells); there are many vesicles discreetly electrodense, with secretory material inside, covered by ribosomes and not (degranulation) communicating with each other.

Intracytoplasmic organelle alterations, together with MP demonstration in all the samples examined, are a very important item since endoplasmic reticulum stress and mitochondrial dysfunction could play a decisive role in human non-transmissible disease (NTD) progression. This gives rise to the hypothesis that plastic environmental pollution is partially responsible for the epidemic of non-communicable disease (NCD) that characterizes the modern world.

Of great concern is the suspicion that MP exposure during critical periods, when adipocytes are differentiating and organs such as the pancreas, liver, and brain are developing, can induce effects that could manifest later in life, often as a full-blown disease.

Developing organisms are sensitive to foreign substances such as plastics in general or MPs. These interferents, like other polluting agents, can lead to abnormal gene expression in tissues, in terms of the number of cells, position, and imbalance between cell types, as well as an impaired organ structure and incorrect hormone signaling. This may lead to an increased susceptibility to disease/dysfunction throughout adult life.

There is ample evidence to support the fact that many chronic diseases, including obesity, diabetes, and metabolic syndromes, may be linked to epigenetic changes in cells and tissues during intrauterine development.

Currently, we are studying placentas collected from uneventful pregnancies to better understand the functional consequences of microplastic detection in developing human beings, especially in relation to the current evolution of the global burden of disease, with increasingly high rates of NCD.

The inconsistency of human thoughts with regard to pollution is explained according to an evolutionist point of view. For millenniums, men considered the environment a hostile setting to conquer and invade. This may be due to the sense of impotence in front of the immense, destructive strength of nature. The description of life before the prevalence of technology in nature could be very similar to what is described by Hobbes in the Leviatano: “a perpetual state of war, nasty, brutal and short.” In the last century, we have witnessed a great evolution, thanks to enormous technological progress. Finally, nature has ceased to be hostile and unpredictable, thus becoming mathematical, linear, and predictable. Mankind now dominates and rules over nature, no longer being a victim of elements and now being able to manipulate nature to satisfy its needs and interests. Mathematics becomes practical, becomes soil and stone, and enters the urban, agricultural, and wild sceneries.

**Figure 1:**
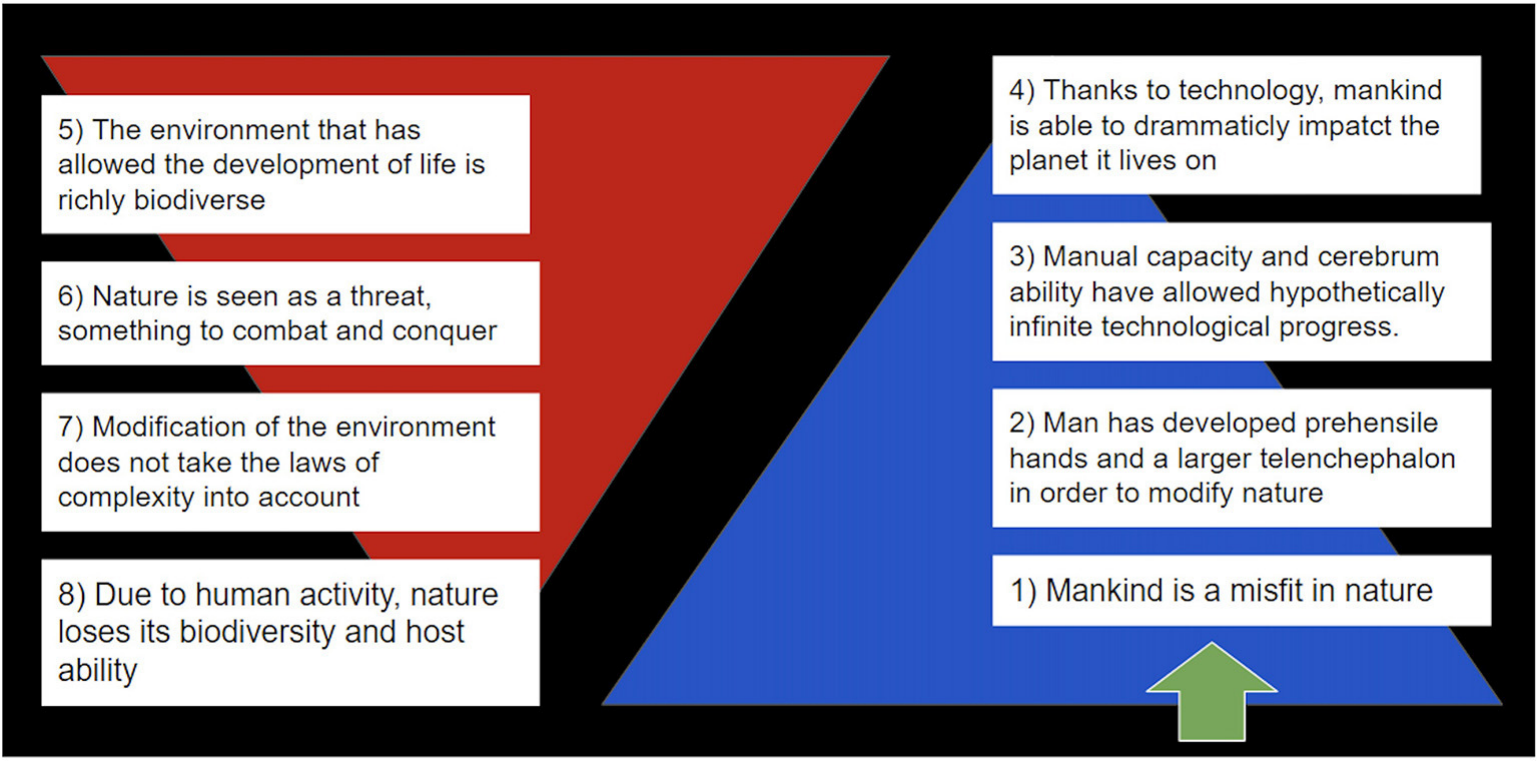
How we have become the greatest competitors in nature and the only beings that do not adapt, but rather modify the environment.

An increasingly big telencephalon (42) together with the opposing thumb (43) has allowed new technological development. Through technology, men stopped adapting themselves to the surrounding world, and nature had to adapt to mankind. This concept is synthetically illustrated in Figure 1.

Although this may have given profit until a century ago, currently human technological capacities are so developed that they could easily lead to the dramatic destruction of nature, an event that is already starting to happen (44). This trend is destined to continue and become worse unless there is a dramatic change in our thought process. This is difficult to picture since the idea that the earth is an enemy to conquer is deeply rooted within the human mind. Respect for biodiversity is not taken into consideration. And just like this, in thousands of years, with an extreme acceleration in the past five decades, men have gone from being victims of nature to being executioners of nature (45). It is necessary to radically change our point of view to preserve our future generations. Nature is not hostile anymore; we have managed to control it. Now, we just need to learn how to respect it.

Another important problem is our perception of the world, which is extremely variable among individuals and not open to considering the long-term consequences of our actions (46).

An extraordinary example of blindness with regard to the future is smoking. It is well-known that there is a latent period between the beginning of smoking and lung cancer development, which may exceed 20 years (47). The fact that an adverse event such as lung cancer might develop in more than 20 years is a difficult concept to consider, thus making it acceptable to continue smoking (48). The blindness mankind shows with regard to the future is the same relative to the environment. Our minds and thoughts are completely occupied by the present moment. Just think about the incorrect use of the word “emergency”, everything seems to be an emergency, even the most chronic and age-old problems such as immigration, war, or plastic pollution (49). FMRI studies suggest that when we imagine our future selves, our brain stops acting as if we were thinking about ourselves while it starts acting as if we were completely different people (50).

In other words, as Jane Mc Gonigal states “your brain acts as if your future self is someone you don't know very well and, frankly, someone you don't care about” (51).

The last problem is the bias of the present, also called hyperbolic discounting: Human decisions are often made to obtain immediate gratification, ignoring the possibility of gain deferred over time. This attitude influences our behavior in at least three important areas of our lives: nutrition, professional life, and savings. In a study conducted by Read and van Leeuwen (52), 74% of participants chose fruits when deciding what to eat the following week, but having to decide what to eat right away, 70% chose chocolates. The same goes for money: We are very willing to take advantage of discounts in the present moment, postponing the worry about more demanding expenses to the future.

This attitude is also a sign of an immediate and insufficient vision of the world, a vision that prevents even expert rulers and politicians from making decisions that are valid in the long run (53). On the contrary, the planet's problems are structural and complex (54), and they must be addressed in this way. The world is a complex place, and the only way to comprehend and change it is to acquire some confidence in the term “complexity”. This will allow us to understand how we reached the threshold of destruction. Understanding how and why we have now entered the Anthropocene and opening our eyes to the deep reasons that lead us to ignore our future and that of the next generations will allow us to intervene in our incorrect behaviors in an adequate way, thus resolving the threats pollution hold in general and not only in pregnancy (55, 56).

In the following two paragraphs, we will briefly discuss the possible solutions to the problem of plastic waste.

## Collective and political responsibility in the use and disposal of plastic

Plastic production and the use of disposable plastic products have a large economic impact from the raw materials used to produce plastic to waste management (13, 57, 58).

The plastics industry represents one of the largest industries in the world, with annual revenues estimated in billions of dollars (59, 60). The development of advanced production technologies and petroleum-derived materials, which are used in the manufacture of plastics, have made it possible to create products that are very cheap to produce and easily available on the market. However, the use of single-use plastic items is significantly contributing to environmental pollution and increased waste generation. This has led several governments to implement restrictions on the use of disposable plastic products, such as plastic bags, straws, plastic cups, and plates (61).

The decision to reduce single-use plastics could lead to an increase in demand for green products, such as biodegradable and compostable products, which can have a positive impact on the economy and the environment, fostering innovation and entrepreneurship. Furthermore, reducing the resources used in the production and management of single-use plastic waste could lead to greater economic efficiency in the long term. While reducing the use of single-use plastics may initially create a negative economic impact on plastic production, it may lead to more sustainable alternative solutions to produce plastic goods in the long run. However due to the large profit margin that the plastic industry currently has, we consider it unlikely that this regulation will happen spontaneously (62), but on the contrary, it should be introduced and regulated by supranational organizations (63, 64). We know that 55% of the world's plastic waste comes from just 20 companies (65). The report found, for example, that only three companies together (ExxonMobil, Dow, and Sinopec) single-handedly account for 16% of global single-use plastic waste. The regulation of these companies should not be impossible.

Another extraordinary step would be to give producers the responsibility for the disposal of plastic waste (66), especially single-use waste, in analogy, for example, to what battery manufacturers do (67).

In this way, much of the problem of the disposal of single-use plastic in the environment would be brilliantly solved because the companies themselves would organize a specific collection and possible recycling, which is now borne by citizens, the end users, who in many places in the world do not have the necessary economic possibilities and the appropriate policy to collect and recycle plastic waste.

The most important responsibility for plastic pollution is currently on the companies that produce it. However, as we will explain in the next paragraph, there is also an individual responsibility, which although quantitatively less important, has a leading educational role.

## Individual responsibility in the use and disposal of plastic

We cannot eliminate the use of plastics from our daily lives, but we can certainly take steps to use and reuse them responsibly (68) by reducing consumption, recycling properly, and finding ways to reuse plastic products, thus minimizing our impact on the environment and moving toward a more sustainable future. There are many ways in which we can responsibly minimize these impacts. To solve the complex problem of plastic pollution, we must incorporate aspects of the importance of responsible consumption in solutions addressing the plastic pollution issue (69).

- One way to responsibly use plastics is to choose products made from recycled or biodegradable materials. Many companies now offer products made from recycled plastics or plant-based materials that provide similar functionality to traditional plastic products but are more sustainable (70).
- Another approach is to reduce our use of plastic altogether. This can be done by opting for products that are not packaged in plastic or by using reusable alternatives to single-use plastic products, such as reusable shopping bags, water bottles, and food containers. By reducing our plastic consumption, we can decrease the amount of plastic waste generated and minimize the environmental impact of plastic production (71).
- In addition to reducing consumption, we can also find ways to reuse plastic products. For example, plastic bags and containers can be washed and reused for storage or other purposes. Some companies even specialize in upcycling plastic waste into new products, such as recycled plastic furniture or clothing (72).
- Lastly, proper disposal of plastic waste is crucial. Plastic products should be recycled whenever possible, and discarded plastic should never be littered in the environment. Many communities have curbside recycling programs, and there are often recycling centers that accept plastic products that cannot be recycled in curbside programs (73).

By taking all these steps, we can minimize the environmental impact of plastic and move toward a more sustainable future.

Finally, by combining individual responsibility, collective political responsibility, and the responsibility of companies that produce plastic (74, 75), we could act to reduce the disposal of a practically indestructible and almost eternal product in the environment, which is considered one of humanity's greatest threats. For this reason, we must quickly become aware of the mental cognitive problems that prevent us from fully understanding and acting accordingly toward the solution of the problem.

## Conclusion

Making a parallel with policies for decarbonization, what should we do to de-plasticize the global economy?

The initial decalogue, to address the problem, could be the following:

1. Sign international agreements to reduce the production of “virgin” plastic.

2. Give responsibility for plastic disposal to plastic producers and not to end consumers.

3. Gradually replace plastic with recyclable material of natural origin.

4. Increase the recycling rate of plastic materials.

5. Ban the sale of mineral water and soft drinks in plastic containers and abolish the use and production of single-use plastics (straws, bags, plates, cutlery, and plastic cups).

6. Use organic material for packaging.

7. Make natural plastic with algae, potatoes, corn, etc.

8. Buy bulk and non-plastic packaged foods.

9. Buy clothing made from natural and non-synthetic materials.

10. Teach all this in schools.

In his essay “The Man in Revolt,” Camus argued that “to be, man must rebel:” Only in this way is it possible to give meaning to one's existence, the reason for the revolt is “… In wanting to serve justice so as not to increase the injustice of the human condition, in striving for clear language so as not to thicken the universal lie and in focusing, despite human misery, on happiness.”

## Author contributions

AR: conceptualization methodology, visualization, supervision, and project administration. AR and AS: original draft preparation and original drawings. AR, AS, DR, EZ, and CD: writing—reviewing and editing. All authors have read and agreed to the published version of the manuscript. All authors contributed to the article and approved the submitted version.
